# Hypothesis: is yeast a clock model to study the onset of humans aging phenotypes?

**DOI:** 10.3389/fonc.2012.00203

**Published:** 2012-12-31

**Authors:** Cristina Mazzoni, Eleonora Mangiapelo, Vanessa Palermo, Claudio Falcone

**Affiliations:** ^1^Department of Biology and Biotechnology “Charles Darwin”, University of Rome “La Sapienza”Rome, Italy; ^2^Pasteur Institute-Cenci Bolognetti Foundation, University of Rome “La Sapienza”Rome, Italy

**Keywords:** aging, apoptosis, cancer, colony, dedifferentiation, keratoses, yeast

## Abstract

In this paper we report the growth and aging of yeast colonies derived from single cells isolated by micromanipulation and seeded one by one on separated plates to avoid growth interference by surrounding colonies. We named this procedure clonal life span, and it could represent a third way of studying aging together with the replicative life span and chronological life span. In this study we observed over time the formation of cell mass similar to the human “senile warts” (seborrheic keratoses), the skin lesions that often appear after 30 years of life and increase in number and size over the years. We observed that similar signs of aging appear in yeast colonies after about 27 days of growth and increase during aging. In this respect we hypothesize to use yeast as a clock to study the onset of human aging phenotypes.

Nowadays*, Saccharomyces cerevisiae *has been widely accepted as a model for the study of aging of multicellular eukaryotes (Kaeberlein, 2010). In fact, in this organism, we can measure the number of mitotic events an individual mother cell can undergo before senescence (Mortimer and Johnston, 1959), referred as replicative life span (RLS). In addition, we can measure the time a non-dividing cell population can remain viable and this is called chronological life span (CLS; Fabrizio and Longo, 2007). RLS has been suggested to be a model for the aging of mitotic tissues, whereas CLS has been likened to the aging of post-mitotic tissues (MacLean et al., 2001) and both can induce apoptosis (Buttner et al., 2006).

Different evolutionary studies are using yeast as a model to investigate on the initial emergence of multicellularity. The formation of multicellular aggregates in liquid cultures can be the result of incomplete cell separation or following the selection of clusters of cells whether by post-division adhesion or by aggregation (Koschwanez et al., 2011; Ratcliff et al., 2012). On solid medium yeast forms colonies after repeated cell divisions.

Here we report the development and aging over 50 days of yeast colonies derived from individual cells of the wild type strain CML39-11A (Mazzoni et al., 2005) isolated by micromanipulation and placed each in one synthetic dextrose (SD) plate. We named this experimental approach clonal life span (ClLS) in that it allows to study the development of clonal cell avoiding the growth interference mediated by the acidic-base pulse generated by surrounding colonies (Váchová and Palková, 2011).

The organization of yeast colonies is ensured by signals transmitted and received by dividing cells within a colony and by chemical alkaline/acid pulse resulting from metabolic activity of colonies growing nearby.

Ammonia signaling is the first non-directed alkaline pulse produced by neighboring colonies and it is followed by a second step leading to acidification of the medium. A second ammonia pulse of higher intensity then occurs and is oriented toward the neighbor colonies. Ammonia signaling results in growth inhibition of the facing parts of near developing colonies (Palková et al., 1997).

The acid phase induces the production of reactive oxygen species (ROS) and other harmful products by all the cells forming the colony and induces apoptosis. The subsequent ammonia signal triggers metabolic changes that allow cells to lower their ROS production (Palková and Vachova, 2006). There is hence a selection of cells within the colony, determined also by the surrounding cells, which limit colony size and development. Cells located at the colony border grow slowly and are healthier compared to the ones located in the center of colonies, which predominantly undergo death.

In the absence of negative growth control exerted by neighboring colonies border cells of an individual colony can divide more and more times, probably without neutralizing ROS. Consequently, colonies might show larger size, peculiar phenotypes, and increased frequency of mutation during aging.

As shown in **Figure 1**, the size of the individually plated colonies increased within 50 days up to 12–13 mm. Interestingly, after 27 days we observed the appearance of some wart-shaped formations. The nature of these excrescences is still unknown, but one can hypothesize that during aging DNA damage and mutation frequency increase (Fabrizio et al., 2005) leading to cells escaping growth control. Although not demonstrated for warts, a similar phenomenon occurs during yeast CLS where the regrowth of few cells, probably adapted mutants generated within aging populations, has been observed (Fabrizio and Longo, 2008). The latter authors suggested that such mutants are reminiscent of cancer cells, which become resistant to apoptosis and duplicate under conditions that are normally not permissive for growth.

**FIGURE 1:**
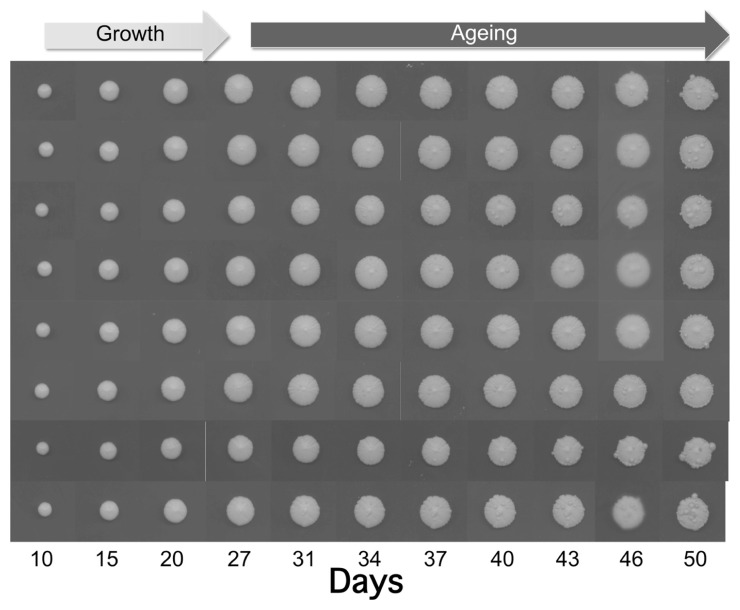
**Growth and aging of colonies derived from of eight yeast cells of the wild type strain CML39-11A (Mat a, ade1-101, his3-δ1, leu2, ura3, trp1-289) isolated by micromanipulation and placed each in one SD (0.67% yeast nitrogen base without amino acids), 2% glucose, and 20 μg/ml of the appropriate nutritional requirements according to the genotype of the strain) plate**. Plates were incubated at 28°C and recorded at the indicated days.

It has been demonstrated that these mutant cells arise from altruistic death program partially mediated by superoxide (Fabrizio et al., 2004). The frequency of this cancer-like regrowth phenotype in yeast liquid cultures is greatly reduced under calorie restriction (CR) and in the presence of mutations in the Tor/Sch9 and Ras/AC/PKA (rat sarcoma/adenylate cyclase/protein kinase A) pathways. For these reasons yeast regrowth has been proposed as a useful phenomenon to study the age-dependent effect of mutations associated with cancer (Madia et al., 2007).

Initially the yeast warts mainly appear in the middle of the colony, where older cells are located, and then they enlarge and propagate along the edge of the whole colony. Anyway, the number of such warts significantly differs from colony to colony suggesting the stochastic nature of these events.

To look closer at the nature of warts, we repeated the experiments extending the aging time up to around 100 days. We compared cell viability and mutation frequency of cells from warts (w) and from the smooth layer (L), picked from the same or different colonies. As shown in **Figure 2A**, w cells showed mainly higher viability compared to L cells. At the same time, we also determined the occurrence of mutations in both w and L cells by measuring the reversion frequency to the prototrophic phenotype of the auxotrophic mutation trp1-289 carried by the CML39-11A strain.

**FIGURE 2:**
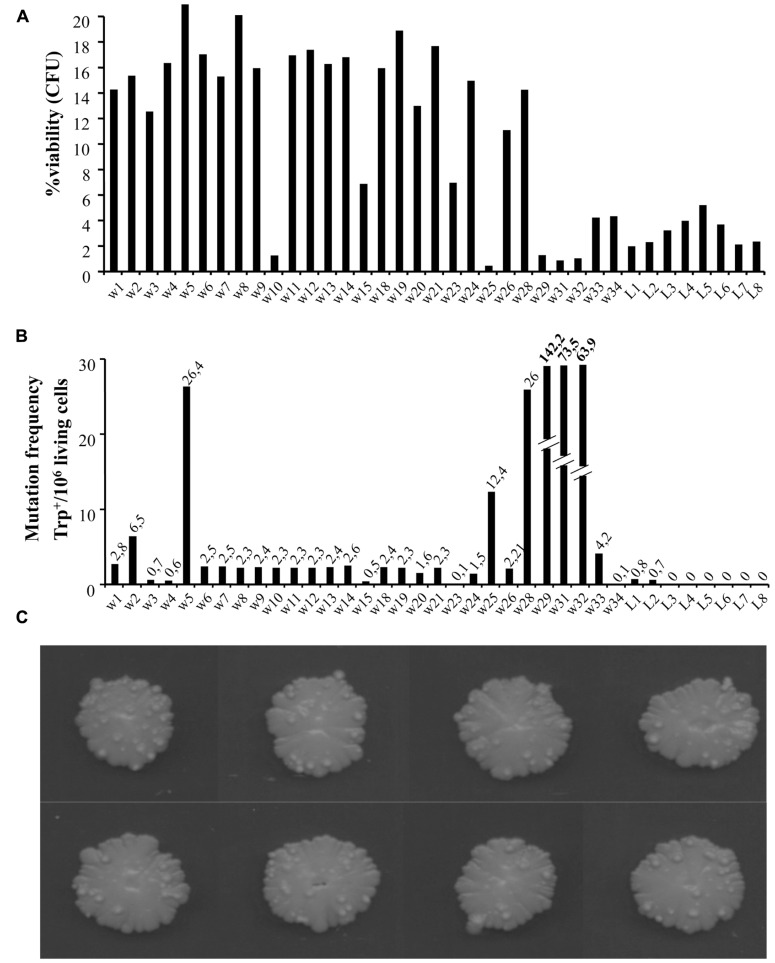
**(A)** Percentage viability of cells picked from single warts (w) or from the smooth layer (L) of colonies after 100 days of growth [shown in **(C)**]. About 1000 cells for each sample were analyzed for their capability to form microcolonies (Palermo et al., 2007). The viability scale was fixed to 20% to better show the lower values. **(B)** Mutation frequency of the same cells measured as Trp^+^ revertants normalized to 10^6^ viable cells.

As shown in **Figure 2C**, the number of warts, compared to 50 days aged colonies, increased significantly, indicating that this phenomenon can occur for long time.

In **Figure 2B** is reported the number of revertants to Trp^+^ phenotype (capability to grow in synthetic medium without tryptophan) normalized to 10^6^ viable cells. As can be seen, the reversion frequency of w cells although heterogeneous, was higher than in L cells, reaching in some cases very high levels (i.e., w29, w31, and w32 values are out of scale).

In cancer cells, nuclear morphology is often altered and nuclei appear bigger and irregular in their contours (Zink et al., 2004). 4′,6-Diamidino-2-phenylindole (DAPI) staining of DNA revealed the presence of round nuclei in cells coming from the smooth surface (**Figure 3A**) as well as in a fraction of w cells (not shown). In addition, part of w cells population showed abnormal nuclei morphology, including bigger dimension, fragmentation, and irregular contours (**Figures 3B**–**D**). Finally, the presence of many cells showing diffused DNA not organized in defined nuclei, together with multinucleated cells, indicates possible defects in cell division.

**FIGURE 3:**
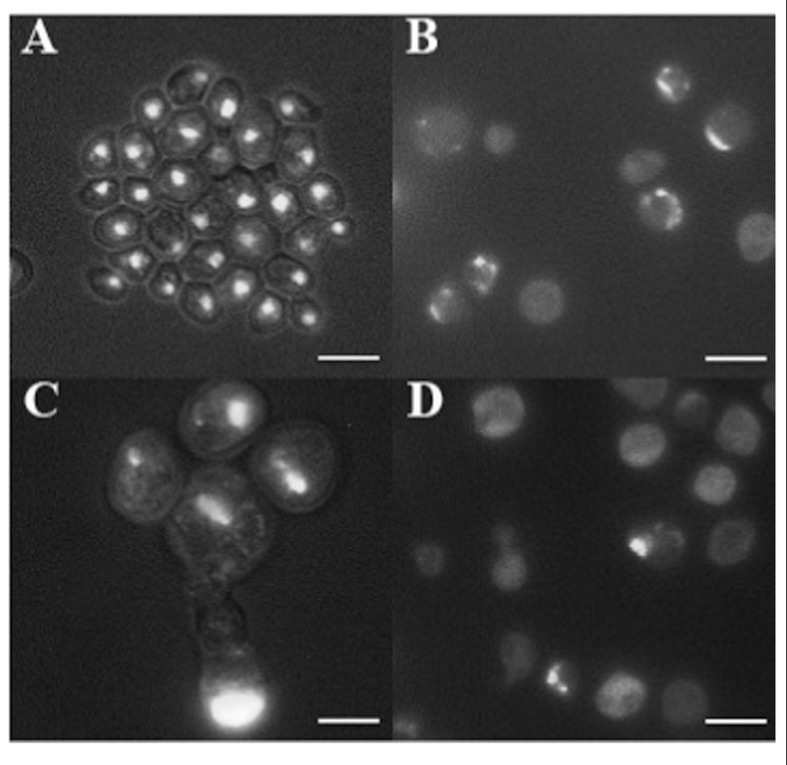
**DAPI staining of cells picked after 100 days from the smooth layer of colonies **(A)** and from three warts (B–D)**. Cells were fixed with 70% ethanol and stained with DAPI at the concentration of 1 μg/ml, and observed by fluorescence microscopy. Cells are shown at the same magnification. Bar, 10 μm.

We like to speculate that these warts could represent the signs of age, just like senile warts [seborrheic keratoses (SKs)], the skin lesions that appear in humans around the age of 30 years and increase in number during aging (Parish and Witkowski, 2005).

Seborrheic keratoses are the most common benign tumors in older individuals and they develop from the proliferation of epidermal cells. Although no specific etiologic factors have been identified, it is known that SK occurs more frequently in sunlight-exposed areas of the body such as the face and the neck (Yeatman et al., 1997). SKs are benign but secondary tumors, and Bowen disease (squamous cell carcinoma *in situ*) or malignant melanoma may occasionally arise within these lesions (Terada, 2010; Böer-Auer et al., 2012).

A similar situation seems to occur in yeast warts, as they can show both normal and cancer-like phenotypes.

Up to now, the patho-mechanisms of SK are not fully understood. Several studies showed that mutations in the fibroblast growth factor receptor 3 (FGFR3) and in the 110 kDa catalytic subunit of the phosphoinositide-3-kinase (PIK3CA) are present in human and mice benign skin tumors (Logie et al., 2005). More recently, some novel insight into the molecular basis of these benign skin lesions came from gene expression analysis study by DNA microarray that identified several upregulated genes, including the oncogenic form δNp63 of the transcriptional regulator p63 (Seo et al., 2012).

Recently, a yeast gene related to p63, NDT80, has been identified in controlling the aging process. In fact, this gene is involved in rejuvenilation of yeast cells during sporulation and yeast cells over-expressing this gene are able to double their lifespan (Unal et al., 2011).

We propose the ClLS approach as an additional method to study the onset of cells escaping growth control that, in turn, can be isolated from colonies for further studies, including genome wide analysis.

The treatment of SK human lesions varies from topical application of 5-fluorouracil (5-FU), cryotherapy, electrodessication, curettage to excisional surgery (Park, 2005; Sand et al., 2008).

Yeast has already been successfully used to screen new compound and/or to assess their mechanisms of action (Cassidy-Stone et al., 2008; La Regina et al., 2009; Palermo et al., 2010, 2011, 2012). In this respect, ClLS can provide a useful tool for the screening of molecules that are able to delay and/or reduce the onset of warts in yeast colonies.

In conclusions, we propose that yeast, by means of the regrowth phenotype during CLS and the appearance of warts during ClLS, can be a valuable model to study the formation of cell mass escaping the control of growth and it will be very interesting to study the nature of genes involved in this phenomenon. Moreover, yeast might represent a nice clock to measure the onset of aging phenotypes in humans.

## Conflict of Interest Statement

The authors declare that the research was conducted in the absence of any commercial or financial relationships that could be construed as a potential conflict of interest.
